# Analysis of Antibiotic Resistance Genes, Environmental Factors, and Microbial Community From Aquaculture Farms in Five Provinces, China

**DOI:** 10.3389/fmicb.2021.679805

**Published:** 2021-06-24

**Authors:** Xu Cheng, Yitong Lu, Yanzhen Song, Ruifang Zhang, Xinyan ShangGuan, Hongzhou Xu, Chengrong Liu, Haixia Liu

**Affiliations:** College of Animal Science and Technology, Northwest A&F University, Xianyang, China

**Keywords:** aquaculture, sediment, antibiotic resistance genes, environmental factors, bacterial community

## Abstract

The excessive use of antibiotics speeds up the dissemination and aggregation of antibiotic resistance genes (ARGs) in the environment. The ARGs have been regarded as a contaminant of serious environmental threats on a global scale. The constant increase in aquaculture production has led to extensive use of antibiotics as a means to prevent and treat bacterial infections; there is a universal concern about the environmental risk of ARGs in the aquaculture environment. In this study, a survey was conducted to evaluate the abundance and distributions of 10 ARGs, bacterial community, and environmental factors in sediment samples from aquatic farms distributed in Anhui (AP1, AP2, and AP3), Fujian (FP1, FP2, and FP3), Guangxi (GP1, GP2, and GP3), Hainan (HP1, HP2, and HP3), and Shaanxi (SP1, SP2, and SP3) Province in China. The results showed that the relative abundance of total ARGs was higher in AP1, AP2, AP3, FP3, GP3, HP1, HP2, and HP3 than that in FP1, FP2, GP1, GP2, SP1, SP2, and SP3. The *sul1* and *tetW* genes of all sediment samples had the highest abundance. The class 1 integron (*intl1*) was detected in all samples, and the result of Pearson correlation analysis showed that the *intl1* has a positive correlation with the *sul1*, *sul2*, *sul3*, *bla*_OXA_, *qnrS*, *tetM*, *tetQ*, and *tetW* genes. Correlation analysis of the bacterial community diversity and environmental factors showed that the Ca^2+^ concentration has a negative correlation with richness and diversity of the bacterial community in these samples. Of the identified bacterial community, Proteobacteria, Firmicutes, Chloroflexi, and Bacteroidota were the predominant phyla in these samples. Redundancy analysis showed that environmental factors (TN, TP, Cl^–^, and Ca^2+^) have a positive correlation with the bacterial community (AP1, GP1, GP2, GP3, SP1, SP2, and SP3), and the abundance of ARGs (*sul1*, *tetW*, *qnrS*, and *intl1*) has a positive correlation with the bacterial community (AP2, AP3, HP1, HP2, and HP3). Based on the network analysis, the ARGs (*sul1*, *sul2*, *bla*_CMY_, *bla*_OXA_, *qnrS*, *tetW*, *tetQ*, *tetM*, and *intl1*) were found to co-occur with bacterial taxa from the phyla Chloroflexi, Euryarchaeota, Firmicutes, Halobacterota, and Proteobacteria. In conclusion, this study provides an important reference for understanding the environmental risk associated with aquaculture activities in China.

## Introduction

Antibiotics are extensively used to prevent and control bacterial infections in medical care, livestock husbandry, and aquaculture (Kümmerer, 2009; Luo et al., 2010). Some of them are also used as growth promoters in aquaculture activities (Chen H. et al., 2018). However, excessive antibiotics and their metabolites would enter the environment, and they might be further absorbed into soil particles and eventually accumulated in sediments because aquatic animals cannot take full advantage of these antibiotics (Kümmerer, 2009). It is worth noting that the abundance of antibiotic resistance genes (ARGs) in soil was associated with the amount of antibiotic residues in the environment (Fahrenfeld et al., 2014), and the ARGs combining with minerals and humus from the environment might exist for a long time (Dang et al., 2017; Hurst et al., 2019; Ma et al., 2019). It is well known that the ARGs have unique biological characteristics, and they could spread by horizontal gene transfer among bacteria of different species and self-amplify among the same species (Guo et al., 2017; Kumar et al., 2017).

The sediments were regarded as an important plot for accumulation and transmission of ARGs (Marti et al., 2014). Shen et al. (2020) reported that several ARGs (*sul1*, *tetG*, *tetW*, *tetX*, and *intl1* gene) were detected in water and sediment of aquaculture farms in Jiangsu Province, China. Chen B. et al. (2018) explored the ARGs in the sediments from bullfrog farms and confirmed that these identified ARGs were able to encode resistance to over 10 categories of antibiotics, such as aminoglycosides, beta-lactams, chloramphenicols, fluoroquinolones, macrolides, polypeptides, sulfonamides, and tetracyclines.

There is a universal concern that the presence of ARGs in sediments is a potential environmental threat (Wang et al., 2015). The antibiotic-resistant bacteria have constituted a huge repository of ARGs in sediments (Martínez, 2008). Once these ARGs have been transferred into the human symbiotic microbes, they would cause great risks of the ecological environment and human health (Smillie et al., 2011; Forsberg et al., 2012). Currently, a study about ARGs in the environment suggested that the existing forms of ARGs largely determine the ways in which these genes are acquired and disseminated among bacterial hosts (Mao et al., 2014). The conception of integron was first proposed by Stokes in 1989 (Stokes and Hall, 1989). It is a key pathway for bacteria to acquire ARGs, which influenced the removal and transfer of ARGs in the bacterial community (Gaze et al., 2011). As one of the most important mobile genetic materials, the integron could capture, rearrange, and express mobile gene cassettes responsible for the spread of ARGs (Zhang X. et al., 2020) and further accelerate the prevalence and transmission of ARGs in the environment (Martinez-Freijo et al., 1998; Cambray et al., 2010).

Previous studies have suggested that the nutrients also promote directly or indirectly the ARGs propagation (Zhao et al., 2017; Zhang J. et al., 2020). Furthermore, the long-time input of nitrogen and phosphorus not only changed the composition of the bacterial community but also drove the propagation of ARGs (Pan et al., 2020). Total organic carbon (TOC) and total dissolved nitrogen (TDN) were potentially important environmental factors, which affected the abundance and diversity of ARGs in urban river systems (Zhou et al., 2017). Moreover, some researches have indicated that bacterial communities shaped the distribution and abundance of ARGs (Huerta et al., 2013; Xiong et al., 2015). These findings suggested that the distribution and prevalence of ARGs are not only related to the use of antibiotics but also affected by many environmental factors. In this study, we aimed to (1) evaluate the relative abundance of 10 ARGs in sediment samples from different aquaculture farms; (2) elucidate the correlation between environmental factors, ARGs abundance, and bacterial community in different aquaculture farms; (3) identify the co-occurrence patterns between ARGs and bacterial taxa.

## Materials and Methods

### Sample Collection

A total of 15 sediment samples were collected from aquaculture ponds distributed in five Chinese provinces, including Anhui (Wuwei, Freshwater aquaculture farm), Fujian (Zhangzhou, Mariculture farm), Guangxi (Qinzhou, Mariculture farm), Hainan (Haikou, Freshwater aquaculture farm), and Shaanxi (Heyang, Freshwater aquaculture farm) between September and October 2019 (Supplementary Figure 1). Three ponds were selected in every aquaculture farm. These aquaculture ponds could produce aquatic products with average 4,000 kg or more per year. Due to the high stocking density, different antibiotics, including sulfonamides, tetracyclines, beta-lactams, and quinolones were used for prophylactic purposes on these farms. No bacterial infections occurred in the sampled ponds in the past year according to our investigation.

Each pond has an area of approximately 900–1,200 m^2^ with a depth of approximately 150–200 cm. The sediments of all sampled ponds have not been cleaned for at least 1 year to ensure that the samples meet the requirements. The samples were collected from water inlets, water outlets, and center areas of each pond (collected the top 10 cm of the sediment) using the CN-100 bottom sampler (Ruibin, China), and the samples of each pond were completely mixed to avoid heterogeneous differences caused by single sampling. After mixing, the sample was sealed in a sterile plastic bag and transported at 4°C to the laboratory. All the samples were divided into two parts and stored at −80°C for further analysis.

### DNA Extraction and Qualitative PCR of Antibiotic Resistance Genes

The genomic DNA was extracted from 0.25 g lyophilized sediment samples using the TIANamp Soil DNA kit (Tiangen, China). All the operations were performed with the product instructions. The quality of DNA was detected using Ultramicro nucleic acid analyzer (Allsheng, China). The PCR amplification was performed to test 10 ARGs (*sul1*, *sul2*, *sul3*, *tetM*, *tetQ*, *tetW*, *qnrB*, *qnrS*, *bla*_OXA_, and *bla*_CMY_) and class 1 integron integrase gene (*intl1*) based on the investigation of antibiotic use in the aquaculture farms of this study. The primers of target genes were synthesized by Sangon Biotech (Shanghai, China), and the primer sequences are shown in Supplementary Tables 1, 2. The PCR conditions were as follows: pre-denaturation at 95°C for 3 min, then 35 cycles of denaturation at 95°C for 30 s, annealing at the specified temperature (Supplementary Table 1) for 30 s, extension at 72°C for 45 s, and a final extension at 72°C for 5 min. PCR products were subsequently detected by agarose gel electrophoresis analysis, and the results are shown in Supplementary Figure 2.

### High-Throughput Sequencing

In order to further analyze the bacterial community composition in the sediment samples, the high-throughput sequencing of bacterial community was analyzed with an Illumina Mi Seq platform at Novogene (Beijing, China). The V3–V4 regions of bacterial 16S rRNA genes were amplified using the primer pair 341F and 806R. The sequencing analysis was processed using QIIME software for 16S rRNA datasets described in a previous literature (Caporaso et al., 2010).

### Quantification of Antibiotic Resistance Genes

PCR products of the target gene were purified with the Universal DNA Purification Kit (Tiangen, China) and ligated into pGM-T vector (Tiangen, China). Subsequently, the pGEM-T vector carrying target gene was transformed into *Escherichia coli* DH5α (Tiangen, China), and the positive clones were acquired after PCR amplification and sequence analysis. Moreover, the recombinant plasmids with target gene were extracted with TIANprep Mini Plasmid Kit (Tiangen, China) and searched for homolog identity with NCBI Blast program. The concentration of recombinant plasmids was checked by Ultramicro nucleic acid analyzer (Allsheng, China), and the standard curves of recombinant plasmids were built with 10-fold serial diluted. The amplification efficiency of all primers ranged from 91.07 to 106.64% with *R*^2^ > 0.99 (Supplementary Table 3). The target gene copy numbers of the sediment samples were calculated with the CT values according to a previous study (Yuan et al., 2019).

The real-time quantitative PCR (Q-PCR) was performed on a LightCycler^®^ 96 instrument (Roche Ltd., Italy) by utilizing SYBR^®^ Green Pro Taq HS Premix (AG, China) according to the manufacturer’s protocol for further analysis. The 10-μl qPCR reaction system contained 2 × SYBR^®^ Green Pro Taq HS Premix (5 μl), 10 μM primer (0.2 μl for each primer, Sangon Biotech, China), RNase-free water (4.1 μl), and DNA samples or standard plasmid (0.5 μl). The amplification condition of qPCR was as follows: initial enzyme activation at 95°C for 30 s, then 40 cycles of at 95°C for 5 s and at 60°C for 30 s.

### Analysis of Environmental Factors

The contents of Ca^2+^, Mg^2+^, and Cl^–^ were determined by ethylene diamine tetraacetic acid (EDTA) volumetric method (Wang et al., 2020). The concentrations of total nitrogen (TN) and total phosphorus (TP) were determined by spectrophotometric method (SEPA, 2002; Trolle et al., 2009), and the standard curve of TN and TP is shown in Supplementary Table 4.

### Statistical Analysis

Pearson correlation analysis was used to analyze the correlation between environmental factors (TN, TP, Cl^–^, Ca^2+^, and Mg^2+^) with the relative abundance of bacterial community and ARGs. Non-metric multidimensional scaling (NMDS) analysis was used to evaluate the difference of bacteria between different sampling sites. Redundancy analysis (RDA) was employed to assess the effects of environmental factors and ARGs on the bacterial community. The co-occurrence between abundance of ARGs and bacterial taxa was analyzed using network analysis based on the Pearson correlation (Li et al., 2015).

Statistical analysis was performed using SPSS 19.0 (IBM, Chicago, IL, United States). The RDA, Mantel test, Pearson correlation, and heatmap were performed in RStudio (v1.2.5019) with several packages, including vegan and pheatmap packages. Co-occurrence networks were constructed by Gephi software (0.9.2; Gephi, WebAtlas, France).

## Results

### Environmental Factors in the 15 Sediment Samples

**FIGURE 1:**
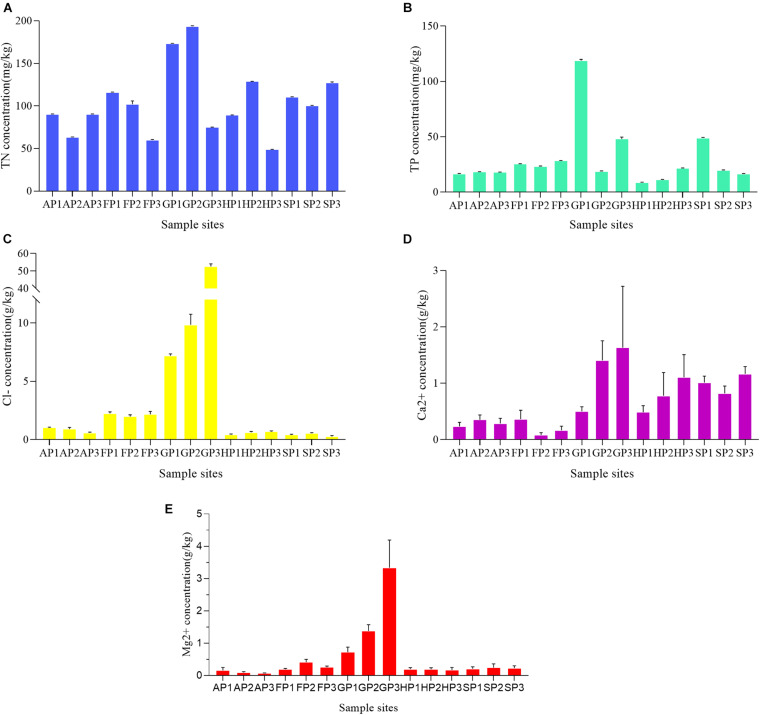
The TN, TP, Cl^–^, Ca^2+^, and Mg^2+^ concentrations **(A–E)** in the 15 sediment samples.

To study the effects of environmental factors on bacterial community in the sediments, the concentrations of five factors (TN, TP, Cl^–^, Ca^2+^, and Mg^2+^) at different sample sites were detected (Supplementary Table 5). Among the sampling sites, TN concentrations ranged from 48.841 ± 0.158 to 193.679 ± 0.45 mg/kg (Figure 1A). The lowest concentration of TN appeared in the sample of HP3 and the highest in the sample of GP2. The TP concentrations were relatively lower in other sampling sites, except GP1 (118.757 ± 0.956 mg/kg), GP3 (48.064 ± 1.373 mg/kg), and SP1 (48.938 ± 0.323 mg/kg) (Figure 1B). The concentration of Cl^–^ has a higher correlation with the aquaculture environment of sampling sites. The Cl^–^ concentration in the sample sites from mariculture farms was generally higher than that of other sampling sites (Figure 1C). The Ca^2+^ concentration of GP3 and FP2 was the highest (1.632 ± 0.889 g/kg) and lowest (0.0800 ± 0.0346 g/kg), respectively (Figure 1D). The concentrations of Mg^2+^ of all sampling sites were generally lower, with the exception of GP1 (0.720 ± 0.128 g/kg), GP2 (1.377 ± 0.159 g/kg), and GP3 (3.334 ± 0.703 g/kg) (Figure 1E).

### Bacterial Community in the 15 Sediment Samples

#### Diversity and Composition of Bacterial Community

With the sequencing of 16S rRNA gene, a total of 61,871 operational taxonomic units (OTUs) were identified from all the 15 sediment samples (Supplementary Table 6). The Rank Abundance curves of OTUs were saturated with all samples (Supplementary Figure 3), which indicated that the abundance and evenness of bacterial community were similar. The indices of ACE, Chao1, Shannon, and Simpson revealed the richness and diversity of the bacterial communities (Supplementary Table 6). The NMDS analysis based on the OTU abundance also indicated that there was no obvious geographic cluster of bacterial communities in different sediment samples (Supplementary Figure 4).

**FIGURE 2:**
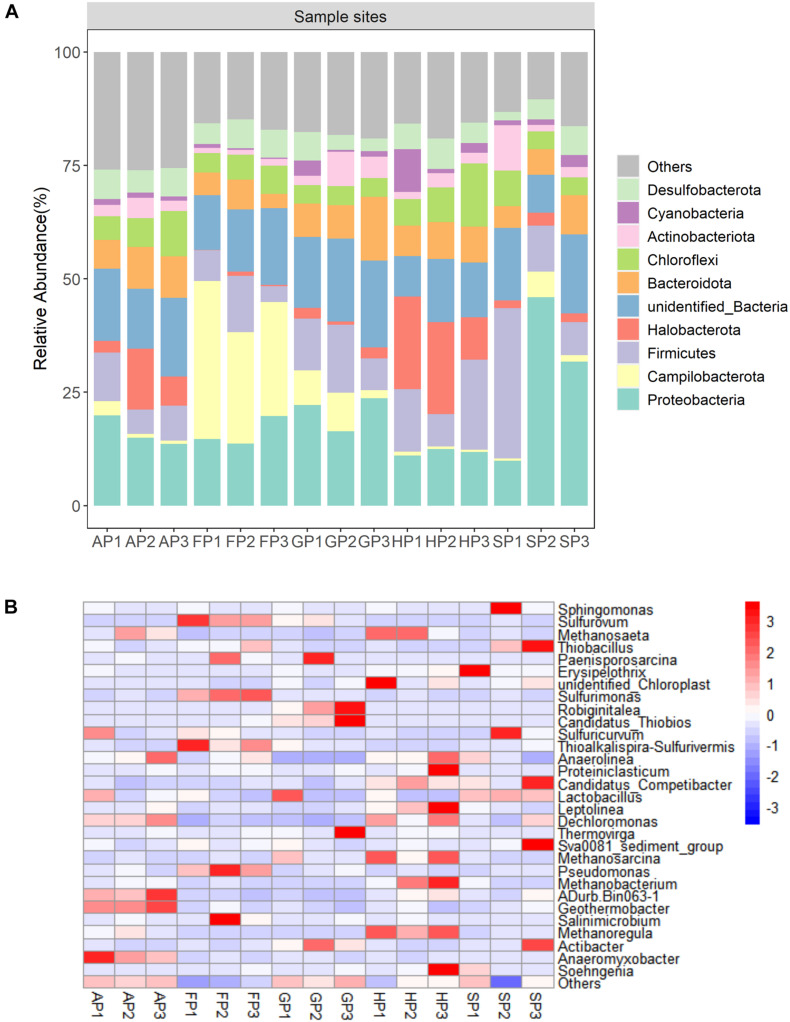
**(A)** Relative abundance of top 10 phyla in the 15 sediment samples. **(B)** The heatmap at the genus level of the 15 sediment samples.

At the phylum level, four predominant phyla (Proteobacteria, Firmicutes, Chloroflexi, and Bacteroidota) were detected in all sediment samples (Figure 2A). The Cyanobacteria has minor abundance, accounting for 0.30–9.39% of total bacterial 16S rRNA sequence libraries. At the genus level, the 16S rRNA sequence libraries detected 30 predominant bacterial genes from the 15 sediment samples. The bacterial community mainly includes *Methanosaeta*, *Sphingomonas*, *Sulfurovum*, and *Thiobacillus* (Figure 2B). In the phylum Proteobacteria, *Dechloromonas*, *Pseudomonas*, *Sphingomonas*, and *Thiobacillus* were the predominant genera of the bacterial community. In the sediment of FP1, FP2, and FP3, *Sulfurovum* and *Sulfurimonas* have a higher abundance (Supplementary Figure 5).

#### Effect of Environmental Factors on the Diversity of the Bacterial Community

The Pearson correlation analysis was used to evaluate the effects of environmental factors on the bacterial community structure. The results indicated that there was no significant correlation between the concentration of TN, TP, Cl^–^, and Mg^2+^ with the richness and the diversity of bacterial community. However, the Ca^2+^ concentration had a significantly negative correlation with the richness and diversity of the bacterial community [OTU (*r* = −0.61, *p* < 0.05), ACE (*r* = −0.65, *p* < 0.05), and Chao1 index (*r* = −0.62, *p* < 0.05)].

### Abundance and Distribution of Antibiotic Resistance Genes

**FIGURE 3:**
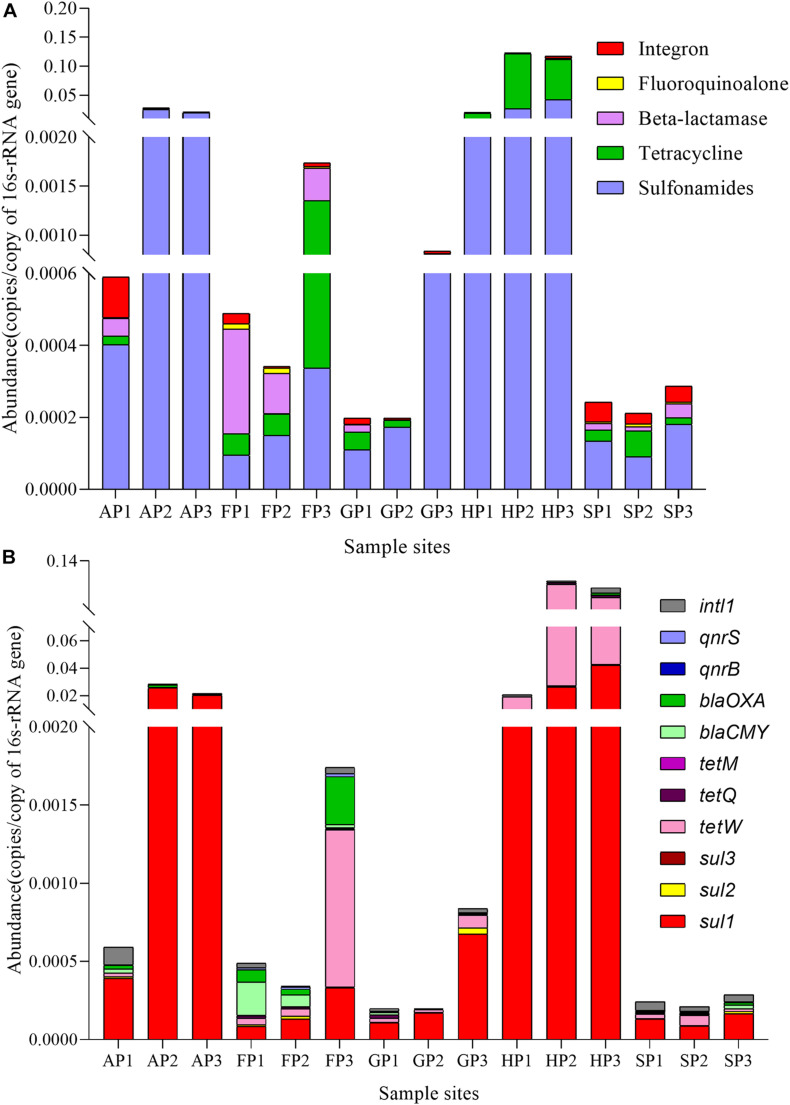
The abundance of antibiotic resistance genes (ARGs) in the 15 sediment samples. **(A)** Five categories. **(B)** 11 kinds.

To analyze the ARGs distribution in different aquaculture farms, 10 tested ARGs and 16S rRNA were investigated. The ARGs abundance was normalized to 16S rRNA genes to compare the difference of ARGs in different samples (Chen et al., 2019). As shown in Figure 3A, the ARGs were classified into four categories (sulfonamide, tetracycline, beta-lactam, and fluoroquinolone resistance genes) and integron. The higher abundance of sulfonamide resistance genes was detected in all these samples, but higher abundance of tetracycline resistance genes was detected in the samples of FP3, HP2, and HP3. The highest abundance of total ARGs was detected in the samples of HP (HP1, HP2, and HP3). The beta-lactam resistance genes have the highest abundance in the samples of FP (FP1, FP2, and FP3).

The distribution of ARGs is shown in Figure 3B. *Sul1*, *sul2*, and *tetM* genes were the highest abundance in all sediment samples, while *sul3* and *qnrS* genes were only detected in the sediments from HP (HP1, HP2, and HP3) and SP (SP1, SP2, and SP3), respectively. Overall, the ARGs levels in different sample sites were obviously different, which could be associated with the different types of antibiotics used in different aquaculture farms.

### Correlations Among Bacterial Community, Antibiotic Resistance Genes, and Environmental Factors

**FIGURE 4:**
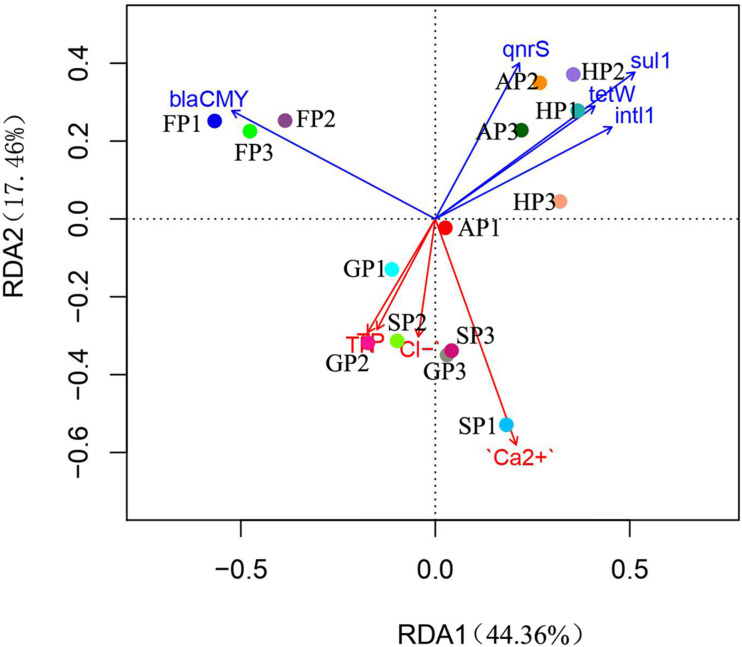
The redundancy analysis (RDA) of environmental factors, antibiotic resistance genes (ARGs), and bacterial communities.

The RDA analysis was performed to further explore the correlation between the bacterial communities of the 15 sediment samples, ARGs abundance, and environmental factors (Figure 4). The weights for variables making up canonical axes of RDA are summarized in Supplementary Table 7. We found that the TN, TP, Cl^–^, Ca^2+^, *sul1*, *bla*_CMY_, *intl1*, *qnrS*, and *tetW* have a significant correlation with the bacterial community in the 15 sediment samples (permutations = 999, *p* < 0.05), which explained 61.82% of overall variation in the bacterial community. The RDA1 and RDA2 explained 44.36 and 17.46% of the total variance, respectively. Positive correlation was found between environmental factors detected in this study and the bacterial community (AP1, GP1, GP2, GP3, SP1, SP2, and SP3), and the bacterial community of the sampling sites showed a negative correlation with ARGs. Moreover, the abundance of ARGs (*sul1*, *tetW*, *qnrS*, and *intl1*) has a much higher correlation with the bacterial community (AP2, AP3, HP1, HP2, and HP3), while *bla*_CMY_ has a stronger correlation with the bacteria of FP (FP1, FP2, and FP3).

Furthermore, the correlation analysis of 10 ARGs showed that there were significant correlations among multiple ARGs (Supplementary Figure 6). Similarly, the *intl1* gene was correlated with the *sul1*, *sul2*, *sul3*, *bla*_OXA_, *qnrS*, and *tetM*, *tetQ*, and *tetW* genes. A Mantel test was also performed to demonstrate whether there were high correlations between the total ARGs and *intl1*. Results showed that a significant correlation (permutations = 999, *r* = 0.8013, *p* < 0.01) was found among the total ARGs and *intl1*.

### Co-occurrence of Bacterial Community and Antibiotic Resistance Genes in Sediment Samples

**FIGURE 5:**
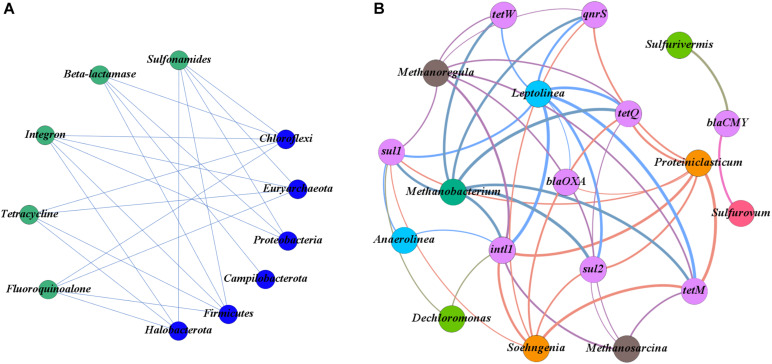
**(A)** Network plots between antibiotic resistance genes (ARGs) and the phyla of the bacterial community. **(B)** Network plots between ARGs and the genera of the bacterial community.

The co-occurrence patterns between ARGs and bacterial community were further analyzed with the network analysis (Figures 5A,B). As shown in Figure 5B, the *intl1* gene was found to co-occur with eight genera of bacteria taxa, followed by *sul1* (7), *sul2* (6), *tetQ* (6), *tetM* (6), *qnrS* (4), *tetW* (3), *bla*_CMY_ (2), and *bla*_OXA_ (2).

At the phylum level of the bacterial community, the main phyla potentially carrying the target ARGs were Chloroflexi, Euryarchaeota, Firmicutes, Halobacterota, and Proteobacteria (Figure 5A). There was a significant co-occurrence pattern in the bacterial taxa of Chloroflexi and eight subtype ARGs (*sul1*, *sul2*, *bla*_OXA_, *qnrS*, *tetW*, *tetQ*, *tetM*, and *intl1*) and a significant co-occurrence pattern in the bacterial taxa of Firmicutes and seven subtype ARGs (*sul1*, *sul2*, *bla*_OXA_, *qnrS*, *tetQ*, *tetM*, and *intl1*) (*p* < 0.05) (Figure 5B). At the genus level of the bacterial community, *Proteiniclasticum*, *Leptolinea*, *Methanobacterium*, and *Methanoregula* were the main potential hosts of ARGs (Figure 5B). Among them, the *Leptolinea* was found to have the most diverse connections with ARGs, including *sul1*, *sul2*, *bla*_OXA_, *qnrS*, *tetW*, *tetQ*, *tetM*, and *intl1* (*p* < 0.05). *Proteiniclasticum* was also detected to carry seven ARGs (*sul1*, *sul2*, *bla*_OXA_, *qnrS*, *tetQ*, *tetM*, and *intl1*) (*p* < 0.05). In addition, *Sulfurovum* and *Sulfurivermis* have simply co-occurred with the *bla*_CMY_ gene encoding resistance.

## Discussion

Aquaculture ponds are regarded as a major reservoir for antibiotic resistant bacteria and ARGs due to the overreliant use of antibiotics (Boran et al., 2013). However, excessive antibiotics and their metabolites have been released into the environment due to the abuse and misuse of antibiotics (Bu et al., 2013; Liu and Wong, 2013). Relevant studies have indicated that antibiotic contamination could lead to the emergence of ARGs in the environment (Qiao et al., 2018; Yang et al., 2018). In this study, we explored the correlation between environmental factors, ARGs, and the bacterial community in different aquatic environments.

Sulfonamides and tetracyclines were used widely in aquatic farms (Luo et al., 2010), the abundance of *sul* and *tet* genes was significantly correlated with the use of the corresponding antibiotics (Gao et al., 2012). Generally, the establishment of ARGs requires selective pressure on antibiotics over a long period. However, once the selective pressure was established, the ARGs would persist and difficult to be eliminated even if the pressure is removed (Pei et al., 2006; Xiao et al., 2016). In this study, a higher abundance of *sul* and *tet* genes in sediment samples was detected, and it was consistent with previous studies that *sul* and *tet* genes were the dominant ARGs in aquaculture water environments (Hoa et al., 2008; Tamminen et al., 2011). Remarkably, the abundance of *sul* gene was higher than that of *tet* gene, except for the sample from FP3. A previous study also indicated that the *sul* gene persists longer than the *tet* gene (McKinney et al., 2010). The *intl1* was one of the mobile genetic element genes and widely existed in Gram-positive and Gram-negative bacteria (Zeng et al., 2019), and it was regarded as an important pollution genetic marker caused by human activity (Gillings et al., 2015). The abundance of *Intl1* gene is a proxy for anthropogenic pollution among many other factors are that they are linked to genes conferring resistance to antibiotics, and *intl1* gene was also closely related to multidrug resistance (MDR). Our study revealed that the abundance of ARGs (*sul1*, *sul2*, *sul3*, *tetW*, *tetQ*, *tetM*, *bla*_OXA_, and *qnrS*) was significantly correlated with the abundance of *Intl1*. It was indicated that *intl1* may play a key role in ARGs proliferation and diffusion from the sediment of aquaculture farms. Moreover, the previous study indicated that there were the co-occurrence patterns among many ARGs in pig farm wastewater (Yang et al., 2020). Correlation analysis in the study also showed that there was a significant positive correlation among the different types of ARGs. The bacteria carrying multiple ARGs could easily obtain the resistance to antibiotics (Trudel et al., 2016); therefore, the potential environmental risk of ARGs should be given attention.

In this study, a significant difference was observed in bacterial communities among different aquatic farms. The dominant phyla were Proteobacteria and Firmicutes in all sediment samples. Similar results were found in pig farms and the sediment of a shrimp farm (Yang et al., 2020; Zeng et al., 2020). Within the Proteobacteria phylum, the *Sphingomonas* was the main compositions of the genus. It was reported that the genome of *Sphingomonas* contains multiple efflux pumps (Jia et al., 2019), suggesting that *Sphingomonas* might better exist in the sediments of aquatic environments. A previous study implied that the physiochemical properties of the environment may influence the bacterial community by affecting the nutrient availability or physiological activity (Li et al., 2019). The present study found that the bacterial communities from AP1, GP1, GP2, GP3, SP1, SP2, and SP3 were significantly correlated with environmental factors. In addition, the concentrations of Mg^2+^, Ca^2+^, and Cl^–^ in the environment influence the composition of the bacterial community (Xu et al., 2018). It is worth noting that the Ca^2+^ has a significant negative correlation with the richness and diversity of bacterial communities. Interestingly, the calcium carbonate was widely used in aquaculture farms, which led to the high accumulation of Ca^2+^ in the sediments. Therefore, the excessive use of calcium carbonate might lead to a decrease in the diversity and richness of bacterial communities in the environment.

The aquatic environment gradually becomes a reservoir of antibiotic-resistant bacteria because of the use and abuse of antibiotics in aquatic farms (Huang et al., 2017). Previous studies certified that some bacterial taxa from Firmicutes were the dominant ARGs-carrying bacteria (Zhang et al., 2021). We also found that the *Proteiniclasticum* and *Soehngenia* from Firmicutes might be the main potential hosts of ARGs, which have strong co-occurrence with *sul1*, *sul2*, *bla*_OXA_, *qnrS*, *tetQ*, *tetM*, and *intl1* genes. Similarly, the *Anaerolinea* and *Leptolinea* from Chloroflexi have strong co-occurrence with ARGs (*sul1*, *sul2*, *bla*_OXA_, *qnrS*, *tetW*, *tetQ*, *tetM*, and *intl1*). However, the *Sulfurovum* from Campilobacterota only has a co-occurrence with *bla*_CMY_, and the Campilobacterota was the dominant phylum in the samples of FP (FP1, FP2, and FP3). Furthermore, there was a stronger co-occurrence between *tetM* gene and six bacterial taxa in these samples. A previous study revealed the similar results in the soil of swine feedlots (Li et al., 2019). It is worth noting that the *tetM* gene was regarded as a detection tool to track and monitor ARGs transport in agricultural systems (Cadena et al., 2018). Our research found that the ARGs have a complex co-occurrence correlation with the bacterial taxa in sediment, which indicated that some bacterial taxa could be resistant to multiple antibiotics in the sediments. Overall, this study indicated that the ARGs in the sediments of aquaculture farms have an impact on the environment and bacterial communities, and we must pay more attention to and take preventive measures.

## Conclusion

The present study indicated that the sulfonamides and tetracycline resistance genes were the predominant ARGs in the sediments of the investigated aquatic farms. Some bacterial taxa from the phyla Chloroflexi, Euryarchaeota, Firmicutes, Halobacterota, and Proteobacteria might be the main potential hosts of ARGs in these aquatic farms. Moreover, the excessive Ca^2+^ might inhibit the diversity and richness of bacterial communities.

## Data Availability Statement

The sequence raw datasets in this study can be found in the NCBI repository (http://www.ncbi.nlm.nih.gov/bioproject/708165).

## Author Contributions

XC and HL played an important role in the conception of the study. CL and YS finished the part of the experiment. RZ and HX organized the original data. YL and XS performed the data analysis. XC wrote the first manuscript. HL edited the final manuscript. All authors contributed to the article and approved the submitted version.

## Conflict of Interest

The authors declare that the research was conducted in the absence of any commercial or financial relationships that could be construed as a potential conflict of interest.
