# Supplementary material for: Analysis of Antibiotic Resistance Genes, Environmental Factors, and Microbial Community From Aquaculture Farms in Five Provinces, China
Source: Front Microbiol. 2021 Jun 24;12:679805. doi: 10.3389/fmicb.2021.679805 (PMC8264556; doi:10.3389/fmicb.2021.679805)
Supplement: Supplementary file 1 [file Data_Sheet_1.docx]

***Supplementary Material***


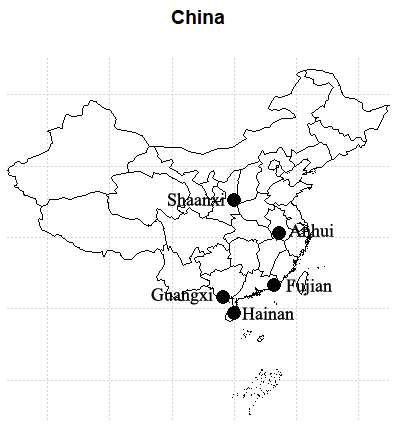


**Supplementary Figure S1**. The location of sample sites. AP1, AP2 and AP3: these sampling sites in Wuwei (Anhui) fresh water farms; FP1, FP2 and FP3: these sampling sites in Zhangzhou (Fujian) sea water farms; GP1, GP2 and GP3: these sampling sites in Qinzhou (Guangxi) sea water farms; HP1, HP2 and HP3: these sampling sites in Haikou (Hainan) fresh water farms; SP1, SP2 and SP3: these sampling sites in Heyang (Heyang) fresh water farms.

**
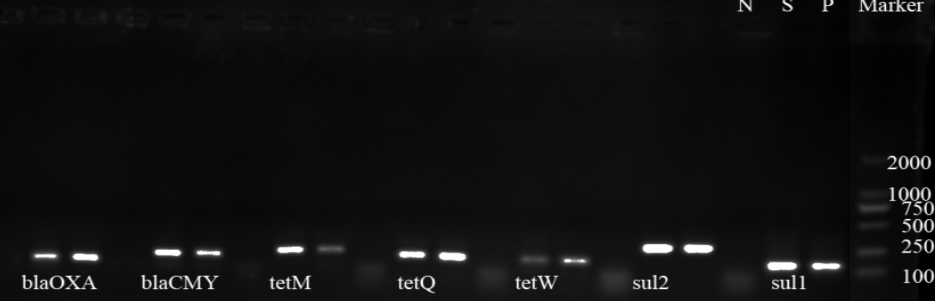
**

**
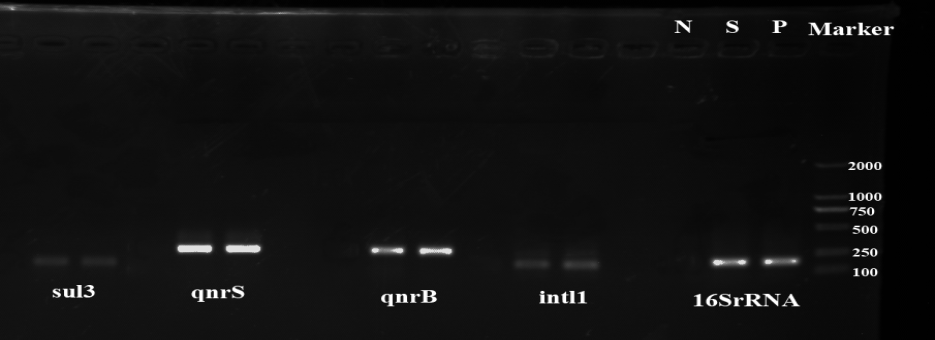
**

**Supplementary Figure S2** The agarose electrophoresis of target primers (N: negative control; S: sample; P: positive control.). The negative control was *E .coli*; the positive control was *E.coli DH5a* with the pGM-T vector which carries target gene.

**
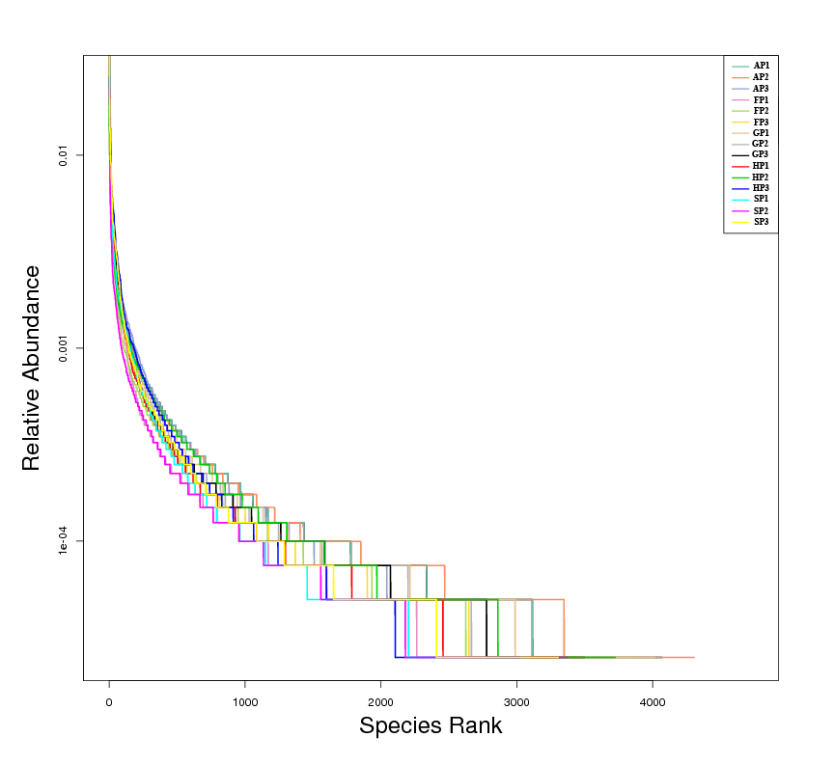
**

**Supplementary Figure S3** The rank curves of microbial communities

**
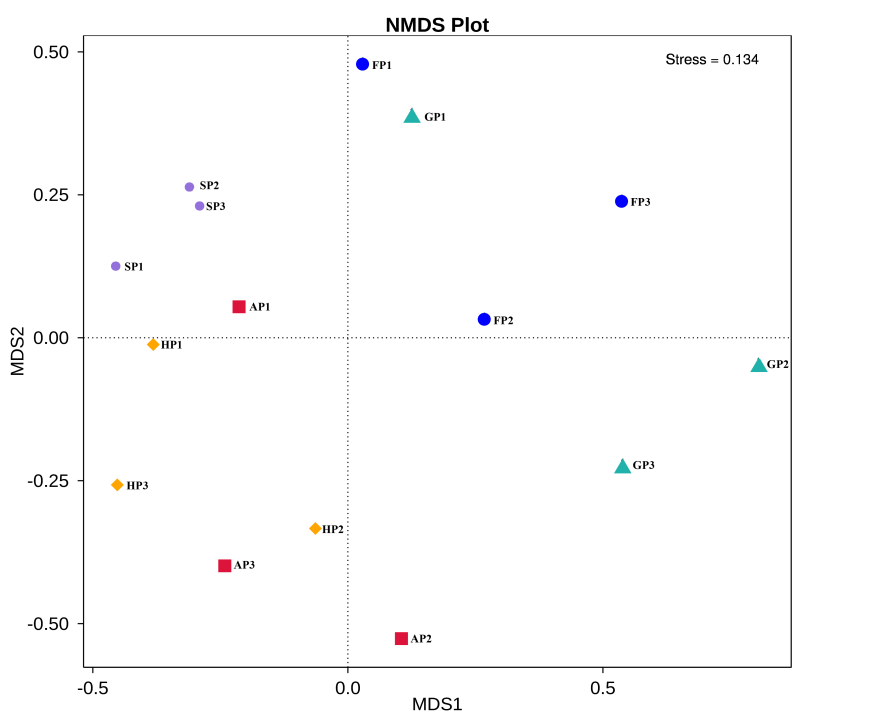
**

**Supplementary Figure S4** Relative abundance of top 100 taxonomic genus in 15 sediment samples

**
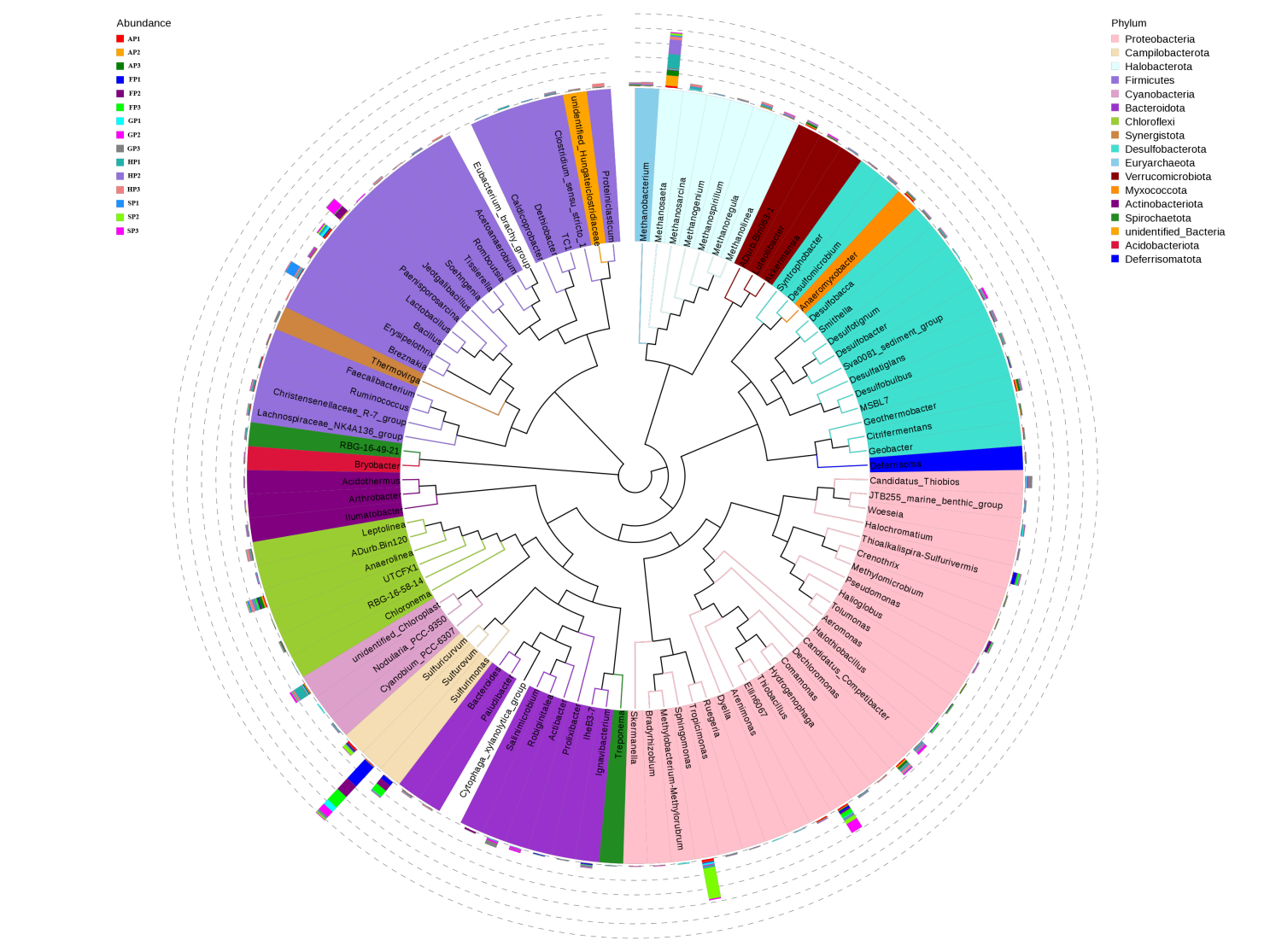
**

**Supplementary Figure S5** Relative abundance of top 100 taxonomic genus in 15 sediment samples

**
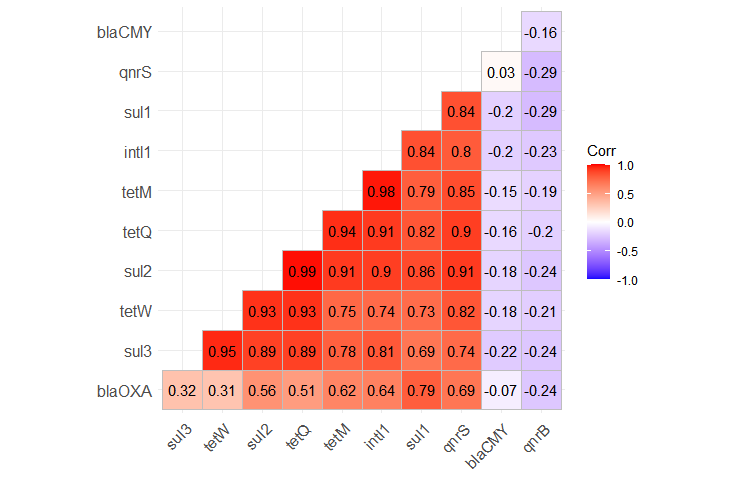
**

**Supplementary Figure S6** Correlation between the abundance of different types of ARGs.Pearson correlation coefficient between 0.50 and 0.79 indicates a moderate correlation, and the r value between 0.80 and 1.00 indicates a strong correlation. The correlation coefficient wit significance p < 0.05 is denoted by background color

| Gene | Primer | Sequence (5’ to 3’) | Amplification Length (bp) | Annealing temperature(℃) | Reference |
| --- | --- | --- | --- | --- | --- |
| *sul1* | FW | CACCGGAAACATCGCTGCA | 158 | 60 | Luo et al. 2010 |
|  | RV | AAGTTCCGCCGCAAGGCT |  |  |  |
| *sul2* | FW | GCGCTCAAGGCAGATGGCATT | 293 | 60 | Frank et al. 2007 |
|  | RV | GCGTTTGATACCGGCACCCG |  |  |  |
| *sul3* | FW | TCCGTTCAGCGAATTGGTGCAG | 127 | 60 | Pei et al. 2006 |
|  | RV | TTCGTTCACGCCTTTCACCAGC |  |  |  |
| *tetQ* | FW | AGAATCTGCTGTTTGCCAGTG | 196 | 60 | Wu et al. 2015 |
|  | RV | CGGAGTGTCAATGATATTGCA |  |  |  |
| *tetM* | FW | CCGTTGGGAAGTGGAATGC | 205 | 60 | Wu et al. 2015 |
|  | RV | TCCGAAAATCTGCTGGGGTA |  |  |  |
| *tetW* | FW | GAGAGCCTGCTATATGCCAGC | 168 | 60 | Aminov et al. 2001 |
|  | RV | GGGCGTATCCACAATGTTAAC |  |  |  |
| *qnrB* | FW | GCGACGTTCAGTGGTTCAG | 148 | 60 | Cummings et al. 2010 |
|  | RV | TGTCCAACTTAACGCCTTGTAA |  |  |  |
| *qnrS* | FW | GACGTGCTAACTTGCGTGAT | 240 | 60 | Marti et al. 2013 |
|  | RV | TGGCATTGTTGGAAACTTG |  |  |  |
| *bla_OXA_* | FW | CGGATGGTTTGAAGGGTTTATTAT | 195 | 60 | Zhu et al. 2013 |
|  | RV | TCTTGGCTTTTATGCTTGATGTTAA |  |  |  |
| *bla_CMY_* | FW | CGTTAATCGCACCATCACC | 171 | 60 | Kurpiel et al. 2011 |
|  | RV | CGTCTTACTAACCGATCCTAGC |  |  |  |
| *intl1* | FW | CCTCCCGCACGATGATC | 280 | 60 | Goldstein et al. 2001 |
|  | RV | TCCACGCATCGTCAGGC |  |  |  |
| *16S rRNA* | FW | CCTACGGGAGGCAGCAG | 178 | 60 | He et al., 2007 |
|  | RV | ATTACCGCGGCTGCTGG |  |  |  |

**Supplementary Table S1** Primer sequences and PCR conditions

**Supplementary Table S2** GenBank accession number of target ARG sequence and corresponding reference strain

| Gene | Accession number | Reference strain: species and source | Percentage identity (%) |
| --- | --- | --- | --- |
| *sul1* | MN699650.1 | Pseudomonas putida strain C4-B08A transposon Tn3, complete sequence; and CRAB-4 (blaCRAB-4), CatB (catB), QnrVC (qnrVC), TniA (tniA), Sul1 (sul1), PncA (pncA), IS91 family transposase, QacE (qacE), AadA24 (aadA24), CmlA (cmlA), AadB (aadB), Tn3, Phosphoribosyl... | 100 |
| *sul2* | [JN088221.1](https://www.ncbi.nlm.nih.gov/nucleotide/JN088221.1?report=genbank&log$=nuclalign&blast_rank=1&RID=84BW6HB1013) | Laribacter hongkongensis strain W10 dihydropteroate synthase (sul2) gene, partial cds | 100 |
| *sul3* | [AY617070.1](https://www.ncbi.nlm.nih.gov/nucleotide/AY617070.1?report=genbank&log$=nuclalign&blast_rank=1&RID=829DT9K801R) | Escherichia coli dihydropteroate synthase (sul3) gene, partial cds | 100 |
| *tetQ* | MT050499.1 | Uncultured bacterium clone tet(Q)_4_NHP_NGR_2017 tetracycline resistance ribosomal protection protein Tet(Q) (tet(Q)) gene, partial cds | 100 |
| *tetM* | [NG_048229.1](https://www.ncbi.nlm.nih.gov/nucleotide/NG_048229.1?report=genbank&log$=nuclalign&blast_rank=73&RID=8295HPZ1016) | Streptococcus pneumoniae PN34 tet(M) gene for tetracycline resistance ribosomal protection protein Tet(M), complete CDS | 100 |
| *tetW* | GU116835.1 | Uncultured organism clone SODw-29 tetracycline resistance protein (tetW) pseudogene, partial sequence | 100 |
| *qnrB* | NG_059323.1 | Citrobacter sp. MGH110 qnrB gene for quinolone resistance pentapeptide repeat protein QnrB85, complete CDS | 100 |
| *qnrS* | [KU644708.1](https://www.ncbi.nlm.nih.gov/nucleotide/KU644708.1?report=genbank&log$=nuclalign&blast_rank=35&RID=82B35ZF701R) | Aeromonas caviae QnrS2 (qnrS2) gene, complete cds | 100 |
| *bla_OXA_* | [NG_056181.1](https://www.ncbi.nlm.nih.gov/nucleotide/NG_056181.1?report=genbank&log$=nuclalign&blast_rank=1&RID=84VX2B45013) | Ralstonia mannitolilytica WCHRM065837 blaOXA gene for OXA-22 family class D beta-lactamase OXA-572, complete CDS | 100 |
| *bla_CMY_* | [NG_073459.1](https://www.ncbi.nlm.nih.gov/nucleotide/NG_073459.1?report=genbank&log$=nuclalign&blast_rank=6&RID=849G1MFT016) | Klebsiella pneumoniae BTS pBTS_01 blaCMY gene for class C beta-lactamase CMY-174, complete CDS | 100 |
| *intl1* | [KC132906.1](https://www.ncbi.nlm.nih.gov/nucleotide/KC132906.1?report=genbank&log$=nuclalign&blast_rank=1&RID=84FMPSD5013) | Uncultured bacterium clone intI1_32 class I integron, partial sequence | 100 |
| *16S rRNA* | [JF908990.1](https://www.ncbi.nlm.nih.gov/nucleotide/JF908990.1?report=genbank&log$=nuclalign&blast_rank=1&RID=82CH380801R) | Uncultured delta proteobacterium clone 91 16S ribosomal RNA gene, partial sequence | 100 |

**Supplementary Table S3** Standard application curve of selected genes

| Genes | Standard curve | R2 | Amplification efficiency (%) |
| --- | --- | --- | --- |
| *sul1* | y = -3.1725x + 38.385 | 0.995 | 106.64 |
| *sul2* | y = -3.2214x + 34.637 | 0.9996 | 104.37 |
| *sul3* | y = -3.5564x + 41.28 | 0.9963 | 91.07 |
| *tetW* | y = -3.2547x + 42.391 | 0.9973 | 102.88 |
| *tetQ* | y = -3.2678x + 36.188 | 0.998 | 102.31 |
| *tetM* | y = -3.2004x + 35.783 | 0.9998 | 105.33 |
| *blaCMY* | y = -3.4216x + 38.425 | 0.9994 | 96.002 |
| *blaOXA* | y = -3.4216x + 38.425 | 0.9994 | 96 |
| *qnrB* | y = -3.509x + 38.374 | 0.9996 | 92.74 |
| *qnrS* | y = -3.181x + 37.171 | 0.9998 | 106.24 |
| *intl1* | y = -3.2373x + 36.067 | 0.9983 | 103.66 |
| *16S rRNA* | y = -3.3155x + 40.295 | 0.9997 | 100.27 |

**Supplementary Table S4** Standard curve of TN and TP

|  | Standard curve | R2 |
| --- | --- | --- |
| TN | y = 0.3955x + 0.126 | 0.9905 |
| TP | y = 0.6481x - 0.0334 | 0.9938 |

**Supplementary Table S5** The environmental factors concentration of these sample sites

| Sites | TN  (mg/kg) | TP  (mg/kg) | Cl^-^  (g/kg) | Ca^2+^  (g/kg) | Mg^2+^  (g/kg) | T4  (℃) |
| --- | --- | --- | --- | --- | --- | --- |
| AP1 | 90.265±0.273 | 16.458±0.317 | 1.0217±0.0348 | 0.235±0.0589 | 0.156±0.0760 | 22.5 |
| AP2 | 63.253±0.332 | 18.336±0.096 | 0.901±0.118 | 0.352±0.0691 | 0.0943±0.0200 | 22.7 |
| AP3 | 90.139±0.372 | 18.001±0.131 | 0.589±0.0437 | 0.285±0.0757 | 0.0699±0.00829 | 22.2 |
| FP1 | 116.14±0.215 | 25.408±0.131 | 2.249±0.105 | 0.363±0.129 | 0.189±0.0243 | 24.4 |
| FP2 | 102.107±3.097 | 23.402±0.192 | 1.972±0.122 | 0.0800±0.0346 | 0.413±0.0723 | 24.1 |
| FP3 | 60.008±0.52 | 28.314±0.167 | 2.171±0.205 | 0.165±0.0588 | 0.254±0.0319 | 24.3 |
| GP1 | 173.241±0.315 | 118.757±0.956 | 7.202±0.116 | 0.501±0.0657 | 0.720±0.128 | 25.4 |
| GP2 | 193.679±0.45 | 18.799±0.298 | 9.848±0.736 | 1.408±0.283 | 1.377±0.159 | 25.5 |
| GP3 | 74.926±0.238 | 48.064±1.373 | 52.619±1.205 | 1.632±0.889 | 3.334±0.703 | 25.2 |
| HP1 | 89.338±0.158 | 8.769±0.221 | 0.415±0.057 | 0.485±0.0945 | 0.198±0.0359 | 26.1 |
| HP2 | 128.782±0.158 | 11.187±0.167 | 0.617±0.0627 | 0.778±0.336 | 0.189±0.0401 | 26.4 |
| HP3 | 48.841±0.158 | 21.679±0.126 | 0.688±0.0437 | 1.104±0.330 | 0.169±0.0663 | 26.3 |
| SP1 | 110.535±0.215 | 48.938±0.323 | 0.404±0.0460 | 1.013±0.0917 | 0.208±0.0487 | 22.3 |
| SP2 | 100.548±0.158 | 19.596±0.333 | 0.546±0.0362 | 0.821±0.106 | 0.244±0.0939 | 22.5 |
| SP3 | 127.307±0.806 | 16.51±0.252 | 0.266±0.0608 | 1.165±0.106 | 0.229±0.0579 | 22.1 |

**Supplementary Table S6** Alpha diversity indices of microbial phylotypes in the sediments of 15 sediment samples

| Sample | OTU | shannon | simpson | chao1 | ACE |
| --- | --- | --- | --- | --- | --- |
| AP1 | 4715 | 10.087 | 0.996 | 4651.544 | 4760.543 |
| AP2 | 4783 | 10.042 | 0.993 | 4826.505 | 4955.092 |
| AP3 | 4112 | 9.833 | 0.996 | 4072.947 | 4219.278 |
| FP1 | 4207 | 8.175 | 0.969 | 4601.715 | 4721.598 |
| FP2 | 4490 | 8.781 | 0.981 | 4985.263 | 5008.036 |
| FP3 | 3986 | 8.981 | 0.986 | 3942.654 | 4074.671 |
| GP1 | 4670 | 9.846 | 0.996 | 4696.133 | 4802.469 |
| GP2 | 3640 | 9.096 | 0.983 | 3731.039 | 3656.692 |
| GP3 | 3913 | 9.395 | 0.993 | 3858.564 | 3950.316 |
| HP1 | 3923 | 8.684 | 0.979 | 3804.667 | 3924.675 |
| HP2 | 4279 | 9.417 | 0.983 | 4140.456 | 4371.803 |
| HP3 | 3472 | 9.214 | 0.994 | 3845.941 | 3889.752 |
| SP1 | 3697 | 8.286 | 0.971 | 3613.543 | 3891.218 |
| SP2 | 4079 | 7.11 | 0.874 | 4507.472 | 4589.573 |
| SP3 | 3905 | 8.994 | 0.988 | 3832.971 | 4049.387 |

**Supplementary Table S7** Weights for variables making up canonical axes of RDA visualized

| Variables | Canonical axes | | | | | |
| --- | --- | --- | --- | --- | --- | --- |
|  | RDA1 | RDA2 | RDA3 | RDA4 | RDA5 | RDA6 |
| *sul1* | 0.43345 | 0.1627 | 0.009921 | -0.016254 | 0.066981 | 0.051291 |
| *blaCMY* | -0.4419 | 0.12 | 0.053827 | -0.001184 | -0.002363 | 0.034786 |
| *tetW* | 0.34673 | 0.1251 | 0.086659 | -0.068715 | 0.083676 | -0.015934 |
| *qnrS* | 0.18279 | 0.1729 | 0.067432 | -0.035234 | 0.108531 | 0.025033 |
| *intl1* | 0.38361 | 0.1019 | 0.133346 | -0.046434 | 0.053985 | 0.041645 |
| TN | -0.1472 | -0.126 | 0.011798 | -0.011511 | -0.027448 | 0.006546 |
| TP | -0.12659 | -0.1225 | 0.012789 | -0.04518 | -0.047224 | 0.02877 |
| Cl^-^ | -0.03673 | -0.1306 | -0.127012 | -0.143521 | -0.015962 | -0.002844 |
| Ca^2+^ | 0.1756 | -0.2505 | -0.039943 | -0.09831 | 0.040137 | 0.010795 |

**Reference**

He, S., Gall, D. L., McMahon, K. D. (2007). "Candidatus Accumulibacter" population structure in enhanced biological phosphorus removal sludges as revealed by polyphosphate kinase genes. *Applied and environmental microbiology*. 73(18), 5865–5874. doi: 10.1128/AEM.01207-07

Luo, Y., Mao, D., Rysz, M., Zhou, Q., Zhang, H., Xu, L., et al. (2010). Trends in antibiotic resistance genes occurrence in the Haihe River, China. *Environmental science & technology*. 44(19), 7220–7225. doi: 10.1021/es100233w

Frank, T., Gautier, V., Talarmin, A., Bercion, R., Arlet, G. (2007). Characterization of sulphonamide resistance genes and class 1 integron gene cassettes in Enterobacteriaceae, Central African Republic (CAR). *The Journal of antimicrobial chemotherapy*. 59(4), 742–745. doi: 10.1093/jac/dkl538

Pei, R., Kim, S. C., Carlson, K. H., Pruden, A. (2006). Effect of river landscape on the sediment concentrations of antibiotics and corresponding antibiotic resistance genes (ARG). *Water research*. 40(12), 2427–2435. doi: 10.1016/j.watres.2006.04.017

Wu, D., Huang, Z., Yang, K., Graham, D., Xie, B. (2015). Relationships between antibiotics and antibiotic resistance gene levels in municipal solid waste leachates in Shanghai, China. *Environmental science & technology*. 49(7), 4122–4128. doi: 10.1021/es506081z

Aminov, R. I., Garrigues-Jeanjean, N., Mackie, R. I. (2001). Molecular ecology of tetracycline resistance: development and validation of primers for detection of tetracycline resistance genes encoding ribosomal protection proteins. *Applied and environmental microbiology*. 67(1), 22–32. doi: 10.1128/AEM.67.1.22-32.2001

Cummings, D. E., Archer, K. F., Arriola, D. J., Baker, P. A., Faucett, K. G., Laroya, J. B., et al. (2011). Broad dissemination of plasmid-mediated quinolone resistance genes in sediments of two urban coastal wetlands. *Environmental science & technology*. 45(2), 447–454. doi: 10.1021/es1029206

Marti, E., Balcázar, J. L. (2013). Real-Time PCR assays for quantification of qnr genes in environmental water samples and chicken feces. *Applied and environmental microbiology*. 79(5), 1743–1745. doi: 10.1128/AEM.03409-12

Zhu, Y. G., Johnson, T. A., Su, J. Q., Qiao, M., Guo, G. X., Stedtfeld, R. D., et al. (2013). Diverse and abundant antibiotic resistance genes in Chinese swine farms. *Proceedings of the National Academy of Sciences of the United States of America*. 110(9), 3435–3440. doi: 10.1073/pnas.1222743110

Kurpiel, P. M., Hanson, N. D. (2011). Association of IS5 with divergent tandem blaCMY-2 genes in clinical isolates of Escherichia coli. *The Journal of antimicrobial chemotherapy*. 66(8), 1734–1738. doi: 10.1093/jac/dkr212

Goldstein, C., Lee, M. D., Sanchez, S., Hudson, C., Phillips, B., Register, B., et al. (2001). Incidence of class 1 and 2 integrases in clinical and commensal bacteria from livestock, companion animals, and exotics. *Antimicrobial agents and chemotherapy*. 45(3), 723–726. doi: 10.1128/AAC.45.3.723-726.2001
